# IL-17A deficiency promotes periosteal bone formation in a model of inflammatory arthritis

**DOI:** 10.1186/s13075-016-0998-x

**Published:** 2016-05-10

**Authors:** Anita T. Shaw, Yukiko Maeda, Ellen M. Gravallese

**Affiliations:** Department of Medicine, Division of Rheumatology, University of Massachusetts Medical School, 364 Plantation Street, Suite 223, Worcester, MA 01605 USA

**Keywords:** Interleukin-17, Osteoblasts, Bone, Wnt signaling, Spondyloarthritis

## Abstract

**Background:**

Interleukin-17A (IL-17A) plays a pathogenic role in several rheumatic diseases including spondyloarthritis and, paradoxically, has been described to both promote and protect from bone formation. We therefore examined the effects of IL-17A on osteoblast differentiation in vitro and on periosteal bone formation in an in vivo model of inflammatory arthritis.

**Methods:**

K/BxN serum transfer arthritis was induced in IL-17A-deficient and wild-type mice. Clinical and histologic inflammation was assessed and periosteal bone formation was quantitated. Murine calvarial osteoblasts were differentiated in the continuous presence of IL-17A with or without blockade of secreted frizzled related protein (sFRP)1 and effects on differentiation were determined by qRT-PCR and mineralization assays. The impact of IL-17A on expression of Wnt signaling pathway antagonists was also assessed by qRT-PCR. Finally, regulation of Dickkopf (DKK)1 expression in murine synovial fibroblasts was evaluated after treatment with IL-17A, TNF, or IL-17A plus TNF.

**Results:**

IL-17A-deficient mice develop significantly more periosteal bone than wild-type mice at peak inflammation, despite comparable severity of inflammation and bone erosion. IL-17A inhibits calvarial osteoblast differentiation in vitro, inducing mRNA expression of the Wnt antagonist sFRP1 in osteoblasts, and suppressing sFRP3 expression, both potentially contributing to inhibition of osteoblast differentiation. Furthermore, a blocking antibody to sFRP1 reduced the inhibitory effect of IL-17A on differentiation. Although treatment with IL-17A suppresses DKK1 mRNA expression in osteoblasts, IL-17A plus TNF synergistically upregulate DKK1 mRNA expression in synovial fibroblasts.

**Conclusions:**

IL-17A may limit the extent of bone formation at inflamed periosteal sites in spondyloarthritis. IL-17A inhibits calvarial osteoblast differentiation, in part by regulating expression of Wnt signaling pathway components. These results demonstrate that additional studies focusing on the role of IL-17A in bone formation in spondyloarthritis are indicated.

## Background

Spondyloarthritis is a debilitating disease in which articular bone erosion occurs and, in addition, new bone is formed at periosteal sites around joints where tendons and ligaments insert, termed enthesophytes. Interleukin-17 (IL-17) contributes to the pathogenesis of inflammation in spondyloarthritis. Isoforms A through F comprise the IL-17 family and both IL-17A and IL-17F are pro-inflammatory, with IL-17A being more widely studied. IL-17 induces the expression of proinflammatory cytokines, including tumor necrosis factor (TNF), IL-1, and IL-6 in stromal cells and macrophages [1, 2], and promotes osteoclastogenesis by upregulating receptor activator of nuclear factor-κB ligand (RANKL) expression on osteoblasts and synovial fibroblasts [3, 4]. IL-17A blockade in inflammatory arthritis has therefore been shown to limit articular bone erosion [5, 6]. IL-17 protein levels are elevated in serum from patients with ankylosing spondylitis compared to healthy controls [7, 8]. Furthermore, mice that develop experimental enthesitis exhibit expansion of lymphocytes that secrete IL-17 [9], and dendritic cells from HLA-B27 transgenic rats produce elevated levels of IL-17 from CD4+ T cells, further supporting a role for IL-17 in inflammation in spondyloarthritis [10].

There is controversy over the effects of IL-17A on bone, and specifically on osteoblast differentiation and function. Both cortical and trabecular bone in femurs of IL-17A-deficient and control C57BL/6 mice exhibit similar bone mineral density, indicating that IL-17A does not significantly alter normal skeletal development [11, 12]. Furthermore, bone volume per total volume (BV/TV), osteoclast number per bone perimeter, and bone formation rate per bone surface (BFR/BS) are similar in IL-17A-deficient and control mice [12]. Osteoblasts are derived from mesenchymal precursor cells, and human mesenchymal stem cells (hMSCs) express the IL-17 receptor [13]. Some studies indicate that IL-17 protects against generalized bone loss by inducing osteoblast differentiation from hMSCs [13, 14] and prevents ovariectomy (OVX)-induced bone loss [15]. Specifically, IL-17 receptor A (IL-17RA)-deficient mice are more susceptible to OVX-induced bone loss compared to controls and have increased leptin levels. IL-17 signaling suppresses adipogenesis and promotes osteoblast differentiation from MSCs, suggesting that IL-17 is bone protective [15]. However, conflicting studies show that IL-17A blockade or IL-17RA deficiency protects from OVX-induced bone loss [16, 17]. IL-17 also inhibits bone regeneration in a rat model of calvarial defect [18], providing additional evidence that IL-17 inhibits osteoblast function.

We therefore sought to determine the effects of IL-17A on osteoblast function in vitro and on osteoblast-mediated bone formation in vivo. We took advantage of the K/BxN serum transfer murine model of inflammatory arthritis (STA), as it has been previously demonstrated that although IL-17A is expressed at low levels, inflammation is not dependent upon IL-17A in this model [19], allowing us to compare differences in the impact of inflammation on bone in the presence or absence of IL-17A. In STA, as in most murine models of inflammatory arthritis including collagen-induced arthritis and antigen-induced arthritis, articular inflammation results in bone destruction; in addition, however, periosteal bone formation occurs reproducibly in all of these models at sites of tendon and ligament insertions, similar to what is observed in spondyloarthritis [20–23]. Thus, STA allows for the direct examination of the effects of inflammation on periosteal bone formation in vivo in the absence of IL-17A.

Here, we demonstrate that IL-17A inhibits osteoblast differentiation and modulates the Wnt signaling pathway in vitro. We further show in vivo that mice deficient in IL-17A develop increased periosteal new bone compared to wild-type controls.

## Methods

### K/BxN serum transfer model of arthritis

Animal procedures were performed in accordance with protocol A-2262 approved by the IACUC at the University of Massachusetts Medical School. KRN T cell transgenic mice (provided by Drs. Benoist and Mathis, Harvard Medical School and Institut de Genetique et de Biologie Moleculaire et Cellulaire, Illkirch, France) were crossed with NOD mice (Jackson) to generate arthritic K/BxN mice [24, 25]. Arthritogenic serum was prepared from 9-week-old K/BxN mice. STA was induced in 12-week-old male IL-17A-deficient mice (Jackson). Absence of IL-17A expression was confirmed by intracellular staining (personal communication, Dr. Keiji Hirota). These mice were backcrossed onto the C57BL/6 background for six generations and C57BL/6J mice (wild-type) were used as controls. Arthritogenic serum, 150 μl per intraperitoneal injection, was administered on days 0, 2, and 7. Ankle thickness was measured as an indicator of inflammation and clinical inflammation was scored as previously described [26].

### Histopathologic analysis

Ankles were fixed in 4 % paraformaldehyde, decalcified and paraffin-embedded. Fifty 5 μm sections were obtained and every ninth and tenth serial section was stained with hematoxylin and eosin (H&E) or tartrate resistant acid phosphatase (TRAP) [27] for histopathologic scoring using previously defined criteria [28].

### Quantitation of periosteal new bone formation

Images of H&E stained sections (#10, 30, and 50) were captured using a Nikon DS-Ri1 camera at the tibia and navicular bones, two sites with reproducible areas of periosteal bone formation. Bone formation area was measured using the NIS-Elements BR software (Nikon). The average area for each site was calculated for each mouse and sites were summed to determine total bone formation area. The average area for each site was then multiplied by 0.2 mm (the distance through sections 10 to 50) to calculate volume. In two mice, only 20 slides were available; therefore, sections #2, 10, and 20 were measured. Total periosteal bone formation volume for each mouse was determined by adding the volumes of bone formation from each site.

### Murine calvarial osteoblast cultures

Calvarial bones of neonatal (P0) C57BL/6J mice (Charles River) underwent three sequential digestions with 0.25 % trypsin (Gibco) and 0.1 % collagenase (Roche) in α-minimum essential medium (α-MEM, HyClone): 8 × 10^4^ cells/well were seeded in 6-well plates and maintained in α-MEM supplemented with 10 % FBS (Atlanta) at 37 °C, 5 % CO_2_. At 80 % confluency (day 0), osteoblast differentiation was initiated with media supplemented with 50 μg/ml L-ascorbic acid (Sigma) and 10 mM β-glycerophosphate (Sigma) with 0, 5, or 50 ng/ml recombinant murine IL-17A (R&D Systems). Samples were performed in duplicate and media was changed three times a week. Osteoblasts were stained with Von Kossa stain using 4 % silver nitrate (Sigma). For cytokine stimulation, calvarial osteoblasts were treated with or without 50 ng/ml IL-17A in duplicate on days 7, 14, and 21 of differentiation for 4, 7, or 12 hours. The calvarial osteoblast experiments were each performed three times using osteoblasts from different neonatal mice. Calvarial osteoblasts were also differentiated in the presence of 50 ng/ml recombinant murine IL-17A plus 1 μg/ml rabbit polyclonal anti-sFRP1 antibody (H-90; Santa Cruz Biotechnology, sc-13939) or rabbit IgG control (Santa Cruz Biotechnology, sc-2027). The anti-sFRP1 antibody was selected based on its effective use at 1 μg/ml in antibody blocking experiments in vitro [29]. Samples were performed in triplicate (days 7 and 14) or quadruplicate (days 21 and 28) and the experiment was repeated twice using osteoblasts from different neonatal mice.

### Assay for Wnt signaling in osteoblasts

Calvarial osteoblasts were isolated from TOPGAL transgenic mice (Jackson) that express a β-galactosidase reporter construct responsive to activation of canonical Wnt signaling, such that expression correlates with Wnt activity, as determined by the mammalian β-galactosidase assay kit (Thermo Scientific) with absorbance measurement at 405 nm. Cells were seeded in 96-well plates at 5000 cells/well. At 80 % confluency (day 0), differentiation media was added with 7.5 ng/ml recombinant murine TNF (R&D Systems) or 50 ng/ml IL-17A. β-galactosidase activity was determined on days 0, 7, 14, 21, and 28 of differentiation.

### Murine fibroblast-like synoviocyte (FLS) culture and cytokine stimulation

FLS were isolated from 8-week-old C57BL/6J mice and cultured as previously described [30]. Cells were used between passages 4 and 6: 5 × 10^4^ cells/well were seeded into 24-well plates in 5 % FBS culture media and treated in triplicate with or without 10 ng/ml TNF, 50 ng/ml IL-17A, or a combination of TNF plus IL-17A for 10 or 24 hours. Four independent experiments were performed.

### RNA isolation and qRT-PCR

Total RNA was isolated from calvarial osteoblasts using the RNeasy Mini kit (Qiagen) and RNase-Free DNase set (Qiagen). Total RNA was isolated from FLS using Trizol (Ambion) and treated with the Turbo DNA-free kit (Ambion) to remove contaminating genomic DNA. For all samples, cDNA was synthesized from 500 ng RNA using the iScript cDNA synthesis kit (BioRad). qRT-PCR was performed on samples in duplicate using iScript SYBR Green RT-PCR mix (BioRad). All primers were purchased from Qiagen. Gene expression was normalized to the housekeeping gene hydroxymethylbilane synthase (HMBS). Data are expressed as the relative gene expression in IL-17A-treated compared to untreated osteoblasts using the 2^-ΔΔCt^ method [31]. For FLS experiments, data are expressed as the relative expression in IL-17A and/or TNF-treated compared to untreated FLS. Data represent the mean ± SD.

### Osteogenesis PCR array

cDNA was synthesized from 500 ng RNA using the RT^2^ First Strand Kit (SABiosciences/Qiagen). The RT^2^ Profiler PCR Array Mouse Osteogenesis (SABiosciences/Qiagen) was performed on calvarial osteoblasts cultured with or without 50 ng/ml IL-17A, assessed at day 28 of differentiation using RT^2^ SYBR Green ROX qPCR master mix (SABiosciences/Qiagen). Data were analyzed using the RT^2^ Profiler PCR Array Data Analysis web portal (http://pcrdataanalysis.sabiosciences.com/pcr/arrayanalysis.php). Data represent the mean ± SD of two independent experiments.

### Statistical analysis

Prism 6.0 (GraphPad) was used for graphing and statistical analysis. The Mann-Whitney test was used to calculate the significance of differences between IL-17A-deficient and wild-type mice in the total volume of periosteal bone formation. Statistical analysis of qRT-PCR data was performed using Student’s unpaired *t* test to determine the significance of differences between treated calvarial osteoblasts or FLS and untreated cells. The mean ± SD of the four independent FLS experiments is reported, with the exception of TNF plus IL-17A treatment at 24 hours for one of four experiments, in which the result was more than three standard deviations higher than the mean and was thus considered an outlier. A *p* value <0.05 was considered statistically significant.

## Results

### IL-17A-deficient mice develop increased periosteal bone in an inflammatory setting

**Fig. 1:**
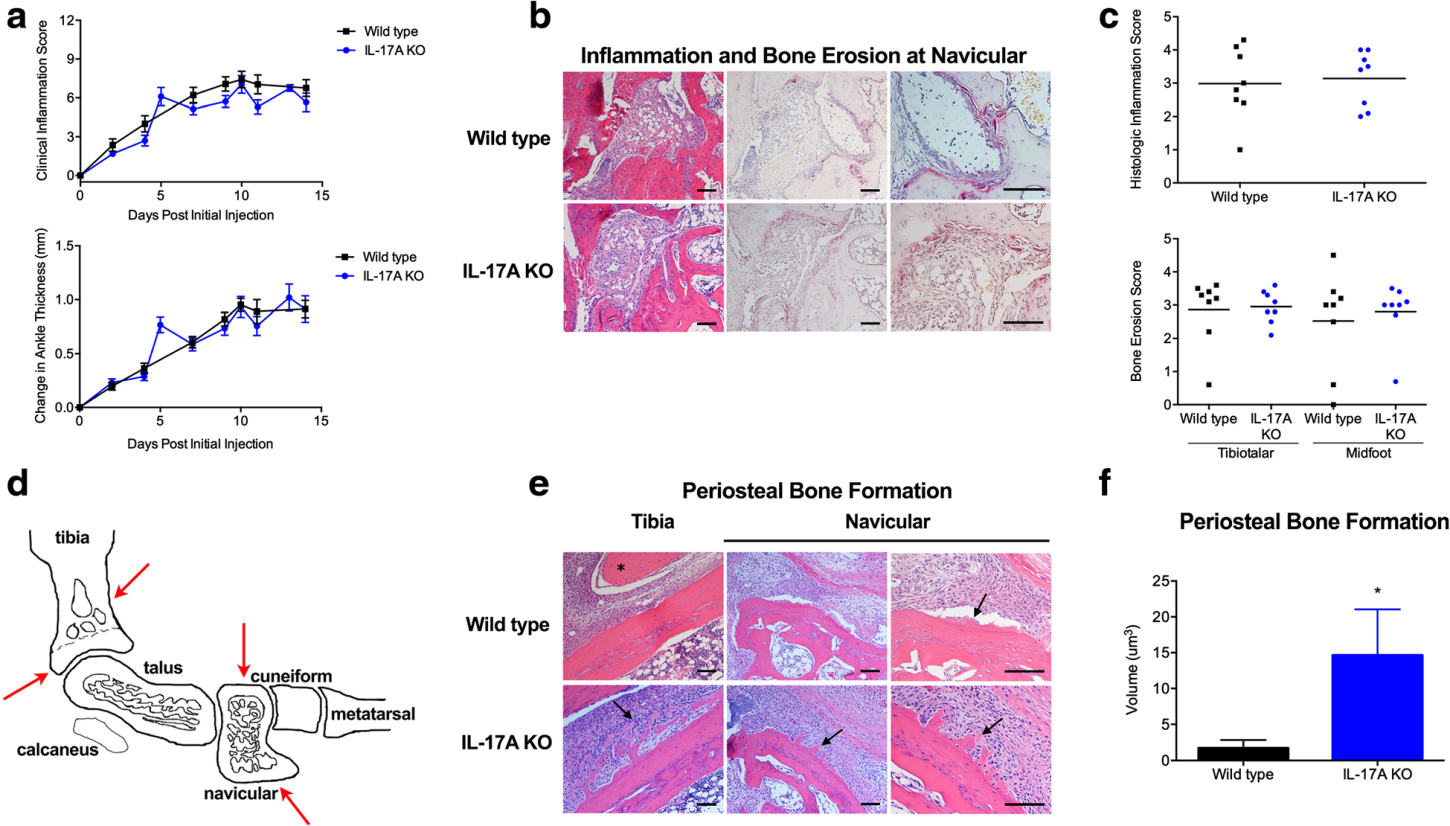
IL-17A-deficient mice induced with serum transfer arthritis develop similar inflammation and bone erosion, but increased periosteal bone. **a** Clinical inflammation scores and change in ankle thickness in IL-17A-deficient (IL-17A knockout (*K*O)) and wild-type mice. Values are the mean ± SEM (n = 24 mice per group, days 0–10; n = 12 mice per group, days 11–14). **b** Representative serial H&E-stained and tartrate-resistant alkaline phosphatase (TRAP)-stained sections of the navicular bone of IL-17A-deficient and wild-type mice. *Scale bar* represents 100 μm. **c** Histopathologic scoring of inflammation and bone erosion of the ankle and midfoot regions in IL-17A-deficient and wild-type mice at peak inflammation. Each *symbol* represents the mean histologic score per mouse (n = 8 mice per group; non-responders with inflammation scores ≤ 0.5 were removed). *Horizontal lines* represent the group means. **d** Schematic of the murine ankle and foot. *Red arrows* indicate reproducible sites of periosteal bone formation in serum transfer arthritis (STA) at the mid and distal tibia and navicular bone. **e** Representative H&E-stained sections of the tibia (*left panels*) and navicular bone (*middle* and *right panels*) of IL-17A-deficient and wild-type mice. *Scale bar* represents 100 μm. *Arrows* indicate areas of periosteal new bone formation. *Tendon inserting on tibia at site of bone formation. **f** Quantitation of the volume of periosteal bone formation at peak inflammation in IL-17A-deficient and wild-type mice. Data represent the mean ± SEM (n = 8 mice per group) (*p < 0.05)

We sought to evaluate the effect of IL-17A deficiency on bone in STA, an animal model in which both articular erosion and periosteal bone formation reliably occur [20, 23]. IL-17A-deficient and wild-type mice were induced with STA, and inflammation, bone erosion, and periosteal bone formation were quantified. IL-17A-deficient and wild-type mice developed similar inflammation (Fig. 1a). Histopathologic scoring of H&E-stained and TRAP-stained ankle sections at peak inflammation (day 10) also revealed a similar extent of bone erosion at the tibiotalar joint and midfoot bones (Fig. 1b, c), confirming that IL-17A does not regulate inflammation or subsequent bone erosion in this inflammatory arthritis model.

The mid and distal tibia and navicular bones are reproducible sites of periosteal bone formation in STA (Fig. 1d). Quantitation of periosteal bone volume revealed a statistically significant difference (Fig. 1e, f) between IL-17A-deficient and wild-type mice at peak inflammation (day 10). Quantitation of periosteal bone area recapitulated these findings (*p* < 0.05, data not shown). These data support the hypothesis that IL-17A may be protective in preventing periosteal bone formation in an inflammatory setting.

### IL-17A suppresses osteoblast differentiation in vitro

**Fig. 2:**
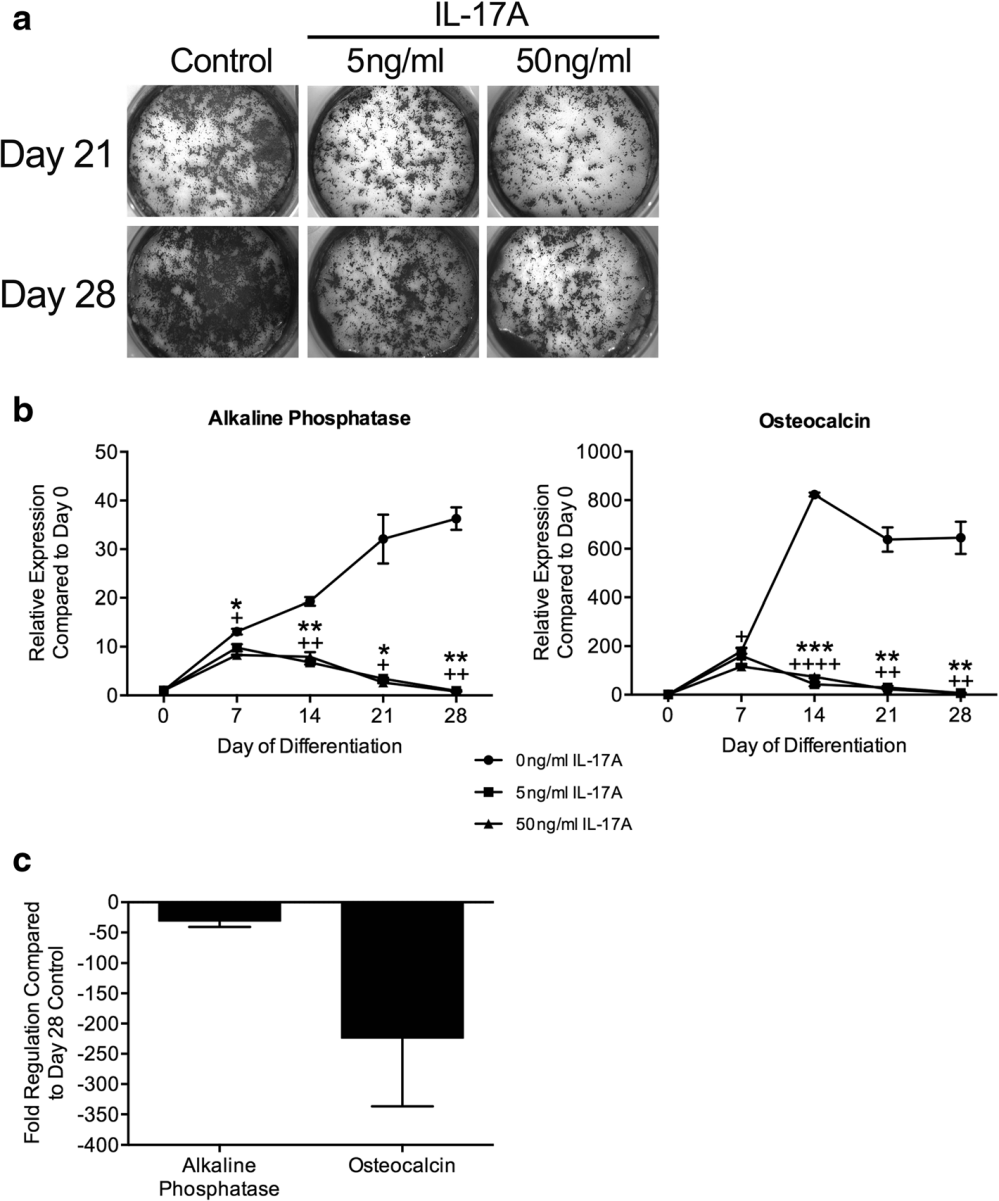
IL-17A inhibits differentiation of cultured calvarial osteoblasts. Osteoblasts are differentiated in the presence or absence of IL-17A (5 or 50 ng/ml). **a** Representative Von Kossa staining at days 21 and 28 of differentiation (n = 3 independent experiments). **b** Representative mRNA expression of alkaline phosphatase and osteocalcin relative to undifferentiated calvarial osteoblasts (day 0) by qRT-PCR over the course of osteoblast differentiation (n = 3 independent experiments) (*p < 0.05, **p < 0.01, ***p < 0.001 for IL-17A at 5 ng/ml; ^+^ *p* < 0.05, ^++^ *p* < 0.01, ^++++^ *p* < 0.0001 for IL-17A at 50 ng/ml). **c** mRNA expression of alkaline phosphatase and osteocalcin by gene array in mature calvarial osteoblasts at day 28, differentiated in the continuous presence of IL-17A (50 ng/ml) compared to day 28 control calvarial osteoblasts

The finding that IL-17A deficiency leads to increased periosteal bone formation in vivo led us to further investigate whether IL-17A directly inhibits osteoblast differentiation. Calvarial osteoblasts were differentiated in the continuous presence or absence of IL-17A (5 or 50 ng/ml). IL-17A inhibited matrix mineralization by mature osteoblasts (days 21 and 28) at 5 ng/ml and more strikingly at 50 ng/ml, as indicated by Von Kossa staining (Fig. 2a). qRT-PCR analysis revealed significant inhibition of expression of alkaline phosphatase, a marker of the mid stage of osteoblast differentiation, and osteocalcin, a marker of the mature, mineralizing osteoblast by osteoblasts differentiated in the presence of IL-17A (5 or 50 ng/ml) (Fig. 2b). Additionally, PCR array analysis confirmed more than 30-fold decrease in alkaline phosphatase and approximately 225-fold decrease in osteocalcin gene expression in osteoblasts differentiated in the presence of IL-17A (50 ng/ml) compared to untreated cells at day 28 (Fig. 2c). These data confirm that IL-17A suppresses calvarial osteoblast differentiation and matrix mineralization in vitro.

### IL-17A modulates Wnt signaling in differentiating osteoblasts

**Fig. 3:**
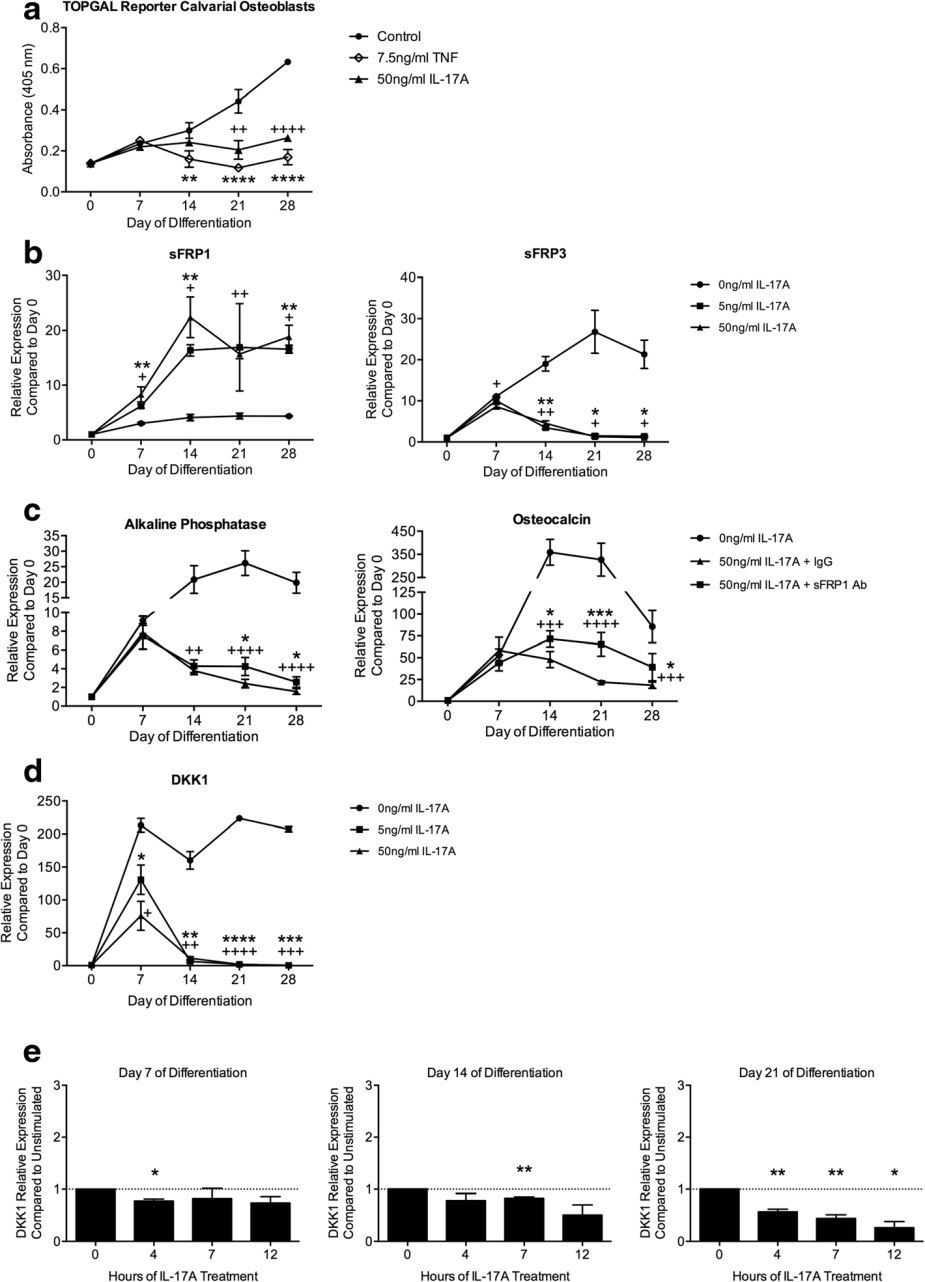
IL-17A modulates Wnt signaling and expression of Wnt signaling antagonists in calvarial osteoblasts. **a** Wnt activity, as determined by β-galactosidase absorbance levels, throughout differentiation of TOPGAL reporter calvarial osteoblasts in the presence or absence of IL-17A (50 ng/ml) or TNF (7.5 ng/ml). Data represent the mean ± SD (triplicate wells) for each condition (**p < 0.01, ****p < 0.0001 for TNF compared to same-day control; ^++^ *p* < 0.01, ^++++^ *p* < 0.0001 for IL-17A compared to same-day control). **b** Representative secreted frizzled related protein (*sFRP*)1, sFRP3 and **d** Dickkopf (*DKK*)1 mRNA expression assessed by qRT-PCR in calvarial osteoblasts cultured in the presence or absence of IL-17A (5 or 50 ng/ml) over the course of differentiation. mRNA expression relative to undifferentiated osteoblasts (day 0) is shown (n = 3 independent experiments) (*p < 0.05, **p < 0.01, ***p < 0.001, ****p < 0.0001 for IL-17A (5 ng/ml); ^+^ *p* < 0.05, ^++^ *p* < 0.01, ^+++^ *p* < 0.001, ^++++^ *p* < 0.0001 for IL-17A (50 ng/ml)). **c** qRT-PCR showing representative expression of alkaline phosphatase and osteocalcin mRNA in differentiating calvarial osteoblasts cultured in the presence of IL-17A (50 ng/ml) plus anti-sFRP1 antibody (*sFRP1 Ab*; 1 μg/ml) or rabbit IgG control (IgG; 1 μg/ml) relative to undifferentiated cells (day 0) (n = 2 independent experiments) (^++^ *p* < 0.01, ^+++^ *p* < 0.001, ^++++^ *p* < 0.0001 for 50 ng/ml IL-17A plus IgG control compared to unstimulated cells; *p < 0.05, ***p < 0.001 for 50 ng/ml IL-17A plus sFRP1 Ab compared to 50 ng/ml IL-17A plus IgG control). **e** Representative DKK1 mRNA expression assessed by qRT-PCR in calvarial osteoblasts (days 7, 14, and 21 of differentiation) treated with IL-17A (50 ng/ml) for 4, 7, or 12 hours. DKK1 expression relative to untreated osteoblasts (0 hours) is shown (n = 3 independent experiments) (*p < 0.05, **p < 0.01)

Activation of the canonical Wnt signaling pathway is essential for osteoblast differentiation. We therefore hypothesized that IL-17A might suppress osteoblast differentiation by inhibiting Wnt signaling. We induced differentiation of calvarial osteoblasts from TOPGAL mice that express a β-galactosidase reporter construct responsive to activation of canonical Wnt signaling, in the presence or absence of IL-17A (50 ng/ml) or TNF (7.5 ng/ml). Wnt signaling, as indicated by β-galactosidase expression, was suppressed in osteoblasts differentiated in the presence of IL-17A (Fig. 3a). TNF also inhibits osteoblast differentiation [32–34] and as expected, the continuous presence of TNF during osteoblast differentiation inhibited Wnt activity in treated cells compared to untreated cells (Fig. 3a). These data support the hypothesis that IL-17A inhibits osteoblast differentiation by suppressing Wnt signaling.

We then sought to identify Wnt pathway components regulated by IL-17A in osteoblasts in vitro. We performed qRT-PCR to determine mRNA expression levels of Wnt antagonist sFRP and DKK family members in calvarial osteoblasts differentiated in the presence or absence of IL-17A (5 or 50 ng/ml). mRNA expression of the Wnt signaling antagonist sFRP1 was induced and mRNA expression of sFRP3 was suppressed by IL-17A at both 5 and 50 ng/ml (Fig. 3b). As a Wnt antagonist, the induction of sFRP1 would result in inhibition of Wnt signaling and inhibited osteoblast differentiation. Importantly, sFRP3 has been shown to promote osteoblast differentiation in bone marrow stromal cells [35]. Therefore, decreased expression of sFRP3 could also result in inhibition of osteoblast differentiation.

To confirm that the induction of sFRP1 by IL-17A contributes to suppressed osteoblast differentiation, we differentiated calvarial osteoblasts in the presence of IL-17A (50 ng/ml) plus a blocking antibody to sFRP1 (1 μg/ml) or control rabbit IgG (1 μg/ml). IL-17A again inhibited osteoblast differentiation as demonstrated by suppressed alkaline phosphatase and osteocalcin mRNA expression in IL-17A-treated osteoblasts plus IgG control compared to untreated osteoblasts (Fig. 3c). Furthermore, osteoblasts treated with IL-17A plus anti-sFRP1 antibody exhibited less suppression of alkaline phosphatase and osteocalcin mRNA expression compared to IL-17A plus IgG control (Fig. 3c), demonstrating that blocking sFRP1 diminishes the inhibition of osteoblast differentiation by IL-17A. Inhibition was not completely rescued solely by blocking sFRP1, suggesting that regulation of expression of other factors modulated by IL-17A, such as sFRP3, may also contribute to the suppression of osteoblast differentiation.

In contrast, mRNA expression of the Wnt antagonist DKK1 was inhibited in the continuous presence of IL-17A (5 or 50 ng/ml) in differentiating calvarial osteoblasts (Fig. 3d). Cytokines, including IL-1 and transforming growth factor-β (TGF-β) can exert different effects on osteoblasts depending upon their stage of differentiation [36]. We thus examined the effect on differentiating calvarial osteoblasts of stimulation with IL-17A (50 ng/ml) at early, mid and late stages of differentiation. IL-17A decreased DKK1 mRNA expression levels compared to levels in untreated cells most significantly in mature osteoblasts (day 21) after 4, 7, or 12 hours of treatment (Fig. 3e). This decrease in DKK1 expression would promote Wnt signaling and osteoblast differentiation.

### IL-17A and TNF synergistically induce DKK1 in FLS

**Fig. 4:**
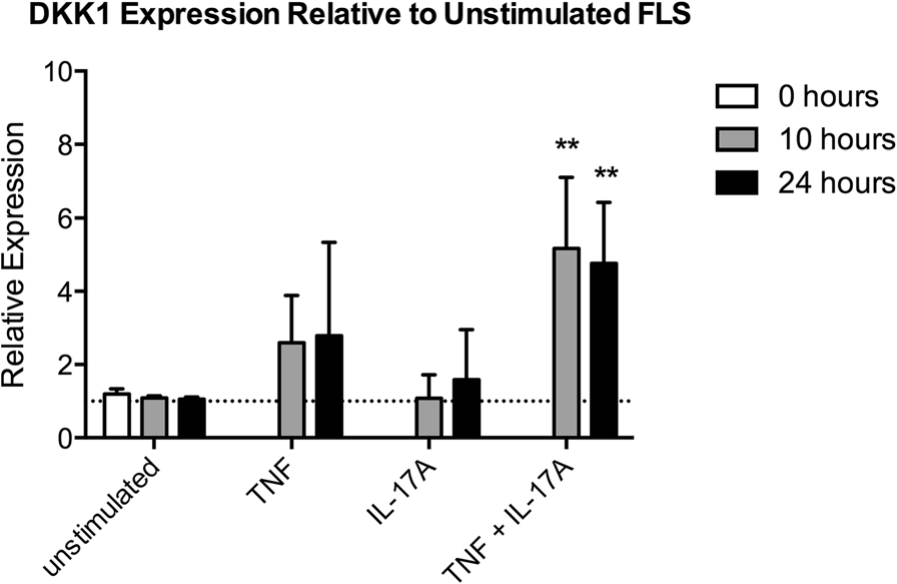
Dickkopf (DKK)1 mRNA expression is synergistically induced by IL-17A plus TNF in fibroblast-like synoviocytes (*FLS*). DKK1 mRNA expression by qRT-PCR in FLS treated with IL-17A (50 ng/ml), TNF (10 ng/ml), or IL-17A plus TNF in combination for 10 or 24 hours. DKK1 expression relative to unstimulated FLS is shown (average of *n* = 4 independent experiments) (**p < 0.01)

Wnt signaling antagonists are also secreted by other cell types, including FLS, and can act distally on osteoblasts. IL-17A and TNF exert a synergistic effect in FLS in rheumatoid arthritis by increasing the secretion of many factors, including IL-6, IL-8 and granulocyte-colony stimulating factor (G-CSF) [1, 37, 38]. We therefore treated murine FLS with TNF (10 ng/ml), IL-17A (50 ng/ml), or both cytokines in vitro. There was a trend towards increased DKK1 mRNA expression by TNF alone. However, IL-17A plus TNF synergistically upregulated DKK1 mRNA expression by approximately fivefold at 10 and 24 hours of treatment (Fig. 4). There was no effect in FLS on mRNA expression of the Wnt antagonists sFRP family members, DKK2, or DKK3 by any treatment (data not shown). The induction of DKK1 by IL-17A plus TNF in FLS could contribute to inhibition of osteoblast differentiation in vivo.

## Discussion

Several lines of evidence support IL-17A as a pathogenic factor that contributes to the inflammatory response in spondyloarthritis and as a promising therapeutic target in rheumatic disease [39–41]. Thus, it is important to understand the impact of IL-17A on bone in an inflammatory setting, with specific attention paid to its effect on bone formation at periosteal and enthesial sites, as this can lead to significant morbidity and disability in spondyloarthritis patients. We therefore sought to determine whether IL-17A has an effect on bone formation at periosteal sites in the inflammatory setting.

IL-17RA comprises part of the receptor complex for many IL-17 isoforms including IL-17A and IL-17F. It has previously been shown that IL-17RA-deficient mice develop less inflammation in the STA model [42], and therefore, the effects on bone independent of inflammation cannot be accurately assessed in IL-17RA deficiency. However, inflammation is not dependent on IL-17A alone in the STA model [19], and therefore we chose to study IL-17A-deficient mice. In this model in which inflammation leads to both articular bone resorption and periosteal bone formation [20, 23], we demonstrated a significant increase in periosteal bone formation in IL-17A-deficient mice. There is also evidence for IL-17A acting as an inhibitor of osteoblast differentiation in vivo. An IL-17A blocking antibody alone has been found not to significantly affect inflammation in arthritic hTNFtg mice [37]. However, blocking antibodies to IL-17A increase serum osteocalcin levels, osteoblast number, and bone formation rates in the tibia, indicating a potential role for IL-17A in bone formation in the long bones.

IL-17A promotes osteoclastogenesis by inducing proinflammatory cytokines and upregulating RANKL; however, reports of its effects on osteoblast differentiation and bone formation are conflicting. We demonstrate that IL-17A inhibits the differentiation of calvarial osteoblasts in vitro. Consistent with our findings, Osta et al*.* observed suppressed DKK1 mRNA expression in hMSCs after 72 hours of treatment with IL-17A, which would promote Wnt signaling and osteoblast differentiation [14]. However, after 6 hours of treatment, IL-17A counteracted the TNF-induced increase in the osteogenic gene bone morphogenetic protein-2 (BMP2), and in conjunction with TNF, induced expression of Schnurri-3, an inhibitor of osteoblast differentiation [43]. Taken together, these data suggest that IL-17A may have differential effects on osteoblast differentiation depending upon the state of differentiation of the osteoblast, and the timing and duration of exposure. In these studies, osteoblasts were differentiated from hMSCs, whereas calvarial osteoblasts were used in our study, which are cells that are already further along the differentiation stage towards the osteoblast lineage. We observed inhibition of osteoblast differentiation by IL-17A in vitro, and these differences could potentially be explained by the use of different precursor cell populations. Our results are in agreement with those of Kim et al*.* who demonstrated inhibition of differentiation of rat calvarial osteoblasts after 14 days of culture with IL-17 in vitro and impaired bone regeneration by IL-17 in a rat model of calvarial defect [18].

We identify regulation of the Wnt signaling pathway as one mechanism by which IL-17A may inhibit osteoblast differentiation and function, as osteoblasts from TOPGAL reporter mice differentiated in the presence of IL-17A exhibited reduced Wnt reporter activity. We studied the effects of IL-17A on Wnt signaling antagonists and found that IL-17A induced sFRP1 and decreased sFRP3 expression, both of which would inhibit osteoblast differentiation. Furthermore, blocking sFRP1 diminished the inhibitory effects of IL-17A on osteoblast differentiation. DKK1 mRNA expression was, in contrast, suppressed in osteoblasts cultured in the presence of IL-17A. However, IL-17A and TNF synergistically induced DKK1 mRNA expression in FLS, which would be predicted to inhibit osteoblast differentiation. The inhibitory effects of IL-17A on osteoblasts were manifested in vivo, as IL-17A-deficient mice developed significantly more periosteal bone in the inflammatory environment.

Patients with spondyloarthritis have excessive bone formation that can occur at entheses (enthesophytes), near joints on the periosteal surface (osteophytes), or in the spine (syndesmophytes). Treatment with non-steroidal anti-inflammatory drugs reduces radiographic evidence of spinal progression in spondyloarthritis after two years of follow up, and long-term treatment with anti-TNF agents may also provide some protection from progression of disease as evidenced by imaging studies [44–46]. Blockade of IL-17A or IL-17RA is a promising therapeutic approach in patients with spondyloarthritis, as it decreases IL-17-induced inflammation. In addition, recent studies demonstrate radiographic evidence of inhibition of the progression of joint structural damage in patients with psoriatic arthritis after a 24-week treatment course with an anti-IL-17A antibody that was maintained through week 52 [47, 48]. Nonetheless, the effects of blocking IL-17A on periosteal bone formation have not yet been evaluated. If IL-17A protects against bone formation by inhibiting osteoblast maturation, blocking IL-17A may potentially be detrimental for patients with spondyloarthritis by promoting and exacerbating bony overgrowth. However, periosteal new bone may not develop if inflammation is well controlled early on by targeting IL-17A. Our finding that periosteal bone formation is increased in the absence of IL-17A in an inflammatory environment stresses the need for additional studies of the interplay between IL-17A, control of inflammation, and bone formation in spondyloarthritis.

## Conclusion

In this report we demonstrate increased bone formation at periosteal sites in the absence of IL-17A in an in vivo inflammatory setting. As IL-17A inhibits calvarial osteoblast differentiation, we propose that IL-17A may play a role in limiting bone formation at inflamed periosteal sites in spondyloarthritis.

## Abbreviations

BMP2: bone morphogenetic protein 2
DKK: Dickkopf
FLS: fibroblast-like synoviocytes
H&E: hematoxylin and eosin
HMBS: hydroxymethylbilane synthase
hMSCs: human mesenchymal stem cells
IL-17A: interleukin-17A
IL-17RA: interleukin-17 receptor A
KO: knockout
OVX: ovariectomy
RANKL: receptor activator of nuclear factor κB ligand
sFRP: secreted frizzled related protein
STA: K/BxN serum transfer arthritis
TNF: tumor necrosis factor
TRAP: tartrate resistant acid phosphatase
α-MEM: α-minimum essential medium
